# Mindfulness for irritable bowel syndrome: protocol development for a controlled clinical trial

**DOI:** 10.1186/1472-6882-9-24

**Published:** 2009-07-28

**Authors:** Susan A Gaylord, William E Whitehead, Rebecca S Coble, Keturah R Faurot, Olafur S Palsson, Eric L Garland, William Frey, John Douglas Mann

**Affiliations:** 1 Program on Integrative Medicine, Department of Physical Medicine and Rehabilitation, School of Medicine, The University of North Carolina at Chapel Hill, Chapel Hill, NC, USA; 2 Department of Medicine, Division of Gastroenterology, School of Medicine, The University of North Carolina at Chapel Hill, Chapel Hill, NC, USA; 3 School of Social Work, The University of North Carolina at Chapel Hill, Chapel Hill, NC, USA; 4 Training & Development, Office of Human Resources, The University of North Carolina at Chapel Hill, Chapel Hill, NC, USA; 5 Department of Neurology, School of Medicine, The University of North Carolina at Chapel Hill, Chapel Hill, NC, USA

## Abstract

**Background:**

Irritable bowel syndrome (IBS), a functional bowel disorder with symptoms of abdominal pain and disturbed defecation experienced by 10% of U.S. adults, results in significant disability, impaired quality of life, and health-care burden. Conventional medical care focusing on pharmacological approaches, diet, and lifestyle management has been partially effective in controlling symptoms. Behavioral treatments, such as cognitive-behavioral therapy and hypnosis, are promising. This paper describes an on-going feasibility study to assess the efficacy of mindfulness training, a behavioral treatment involving directing and sustaining attention to present-moment experience, for the treatment of IBS.

**Methods/Design:**

The study design involves randomization of adult women with IBS according to Rome II criteria, to either an eight-week mindfulness training group (based on a Mindfulness-based Stress Reduction [MBSR] format) or a previously validated IBS social-support group as an attention-control condition. The primary hypothesis is that, compared to Support Group participants, those in the Mindfulness Program will demonstrate significant improvement in IBS symptoms as measured by the IBS Symptom Severity Scale [1].

**Discussion:**

214 individuals have been screened for eligibility, of whom 148 were eligible for the study. Of those, 87 were enrolled, with 21 withdrawing after having given consent. 66 have completed or are in the process of completing the interventions. It is feasible to undertake a rigorous randomized clinical trial of mindfulness training for people with IBS, using a standardized MBSR protocol adapted for those experiencing IBS, compared to a control social-support group previously utilized in IBS studies.

**Trial Registration:**

Clinical Trials.gov Identifier: NCT00680693

## Background

Irritable bowel syndrome (IBS) is a functional bowel disorder characterized by symptoms of abdominal pain or discomfort associated with disturbed defecation [2], experienced by about 10% of the U.S. adult population (14% women, 8% men), and resulting in disability, impaired quality of life, and health-care burden [3-5]. Conventional medical care for IBS has focused on diet and lifestyle management (e.g. exercise and stress reduction), behavioral treatments, and a range of pharmacological approaches, particularly antispasmodics [6]. Pharmacological therapies, which often bear significant costs and side effects, may relieve symptoms temporarily but seldom correct underlying causes. Behavioral treatments, such as cognitive-behavioral therapy and hypnosis, have shown positive results, reflecting the importance of a biopsychosocial perspective.

Mindfulness meditation is a unique behavioral technique which involves the intentional self-regulation of attention by learning to attend to present-moment experience and letting go of cognitive fixation on thoughts of past and future, thus inducing a non-evaluative metacognitive state. Mindfulness practice has been shown to reduce symptoms of stress and pain and ameliorate the symptoms of fibromyalgia and depression [7-11]. There is little research, and no well controlled clinical trials, on its efficacy specifically for IBS. One small, uncontrolled pilot study at UNC found decreased symptom severity in patients with IBS following an eight-week mindfulness-training program [12].

A study of mindfulness for treatment of IBS is warranted for the following reasons: 1) mindfulness training has been shown to be associated with significant symptom reduction in disorders in which both pain and stress are prominent; 2) the techniques of mindfulness can be taught effectively in groups; 3) mindfulness programs are taught as wellness programs, with participants typically reporting positive changes in life-style in addition to improved stress and pain management; 4) as wellness programs, mindfulness can be taught safely and effectively by instructors who may be unlicensed in other disciplines but well-trained in mindfulness; 5) participants report high levels of satisfaction and drop-out rates are low.

Mindfulness meditation may be uniquely suited to treat the underlying psychological symptoms associated with IBS: these include an increased sensitivity to pain from intestinal distention [13] that is correlated with anxiety about the significance of these sensations [14] and selective attention to gastrointestinal sensations [15]. Indeed, recent cognitive neuroscience investigations have found that mindfulness meditation induces alterations in the activation of neural circuits implicated in neurovisceral awareness, interoception (sensitivity to internal stimuli), and self-regulation [16,17]. Additionally, mindfulness has been shown to improve ability to control attentional processes [18-21]. Given these findings, it seems plausible that mindfulness may exert a salutary effect on irritable bowel symptomatology and perhaps even the underlying mechanisms of the syndrome.

In this paper, we describe an ongoing feasibility study to assess the efficacy of a program of mindfulness-based stress and pain management (Mindfulness Program) for treatment of IBS-related symptoms in women. The study includes baseline assessments in patients with documented IBS with subsequent randomization to one of two treatment arms: 1) Mindfulness Program (the intervention group); and 2) an IBS Support Group (the control group) previously validated as an effective placebo-control condition in a randomized controlled trial of cognitive behavior therapy for the treatment of IBS [22]. Outcomes are evaluated at the end of treatment and at 3, 6, and 12 months follow-up.

The study's primary aim is to determine the feasibility of developing a clinical trial comparing effectiveness of a Mindfulness Program with an IBS Support Group in reducing the severity of symptoms in women with IBS. The primary hypothesis is that, compared to those in the Support Group, those in the Mindfulness Program will demonstrate significant improvement in IBS symptoms, as measured by the IBS Symptom Severity Scale [1].

Aim two is to identify appropriate secondary outcomes in the Mindfulness Program and IBS Support Groups, including psychological symptoms, coping strategies, and quality of life. We hypothesize that, compared with those in the Support Group, those in the Mindfulness Program will experience significant reductions in anxiety, depression, anger, and maladaptive coping strategies, as well as improvements in quality of life and effective occupational functioning. Analysis of these variables will lead to better estimates of effect sizes and powering of future studies, and will guide the selection of outcome measures and the design and analyses for those studies.

A third aim is to compare two currently available process measures of mindfulness – the Five Facet Mindfulness Questionnaire (FFMQ) [18-21] and the Toronto Mindfulness Scale (TMS) [23], specifically testing: a) how well the FFMQ vs. the TMS are able to detect any differences in treatment groups; and b) whether either of these scales can discriminate those assigned to the Mindfulness Program who are ranked as more successful at learning and practicing mindfulness from those who are less successful.

## Methods/Design

**Figure 1:**
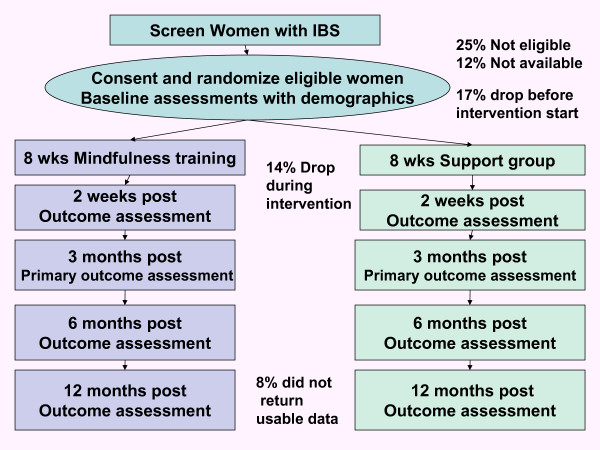
**Overall Design and Subject Flow**.

Figure 1 illustrates the overall design and subject flow through the study. Study procedures and consent forms were reviewed and approved by the Institutional Review Board of The University of North Carolina at Chapel Hill (UNC).

### Hypotheses

The primary hypothesis is that, compared to those in the Support Group, those in the Mindfulness Program will demonstrate significant improvement in IBS symptoms, as measured by the IBS Symptom Severity Scale [1]. Secondary hypotheses are that, compared with those in the Support Group, those in the Mindfulness Program will experience significant reductions in anxiety, depression, anger, and maladaptive coping strategies, as well as improvements in quality of life and effective occupational functioning.

### Eligibility

Included in this trial are women at least 18 years old who have a medical diagnosis of IBS and who also meet the Rome II criteria for IBS [24]. Subjects must be able to understand English and be willing and able to document regularly their IBS symptoms and medication use as well as complete the assessment instruments. Subjects must be willing to attend 8 weekly 2-hour Mindfulness Program or Support Group sessions plus a half-day Saturday session. All subjects are informed that they should continue to receive usual care from their primary care or specialist physicians and that no specific recommendations for changes in medications for IBS or other medical issues will be made by the research team.

Excluded are subjects with the following conditions: 1) diagnosis of mental illness with psychotic features; 2) a history of an inpatient admission for psychiatric disorder within the past two years; 3) a history or current symptoms of inflammatory bowel disease or gastrointestinal malignancy; 3) active liver or pancreatic disease; 4) uncontrolled lactose intolerance; 5) celiac disease; 6) a history of abdominal trauma or surgery involving gastrointestinal resection; or 7) pregnancy or intention to become pregnant during the study.

### Recruitment

Patients under the care of the UNC Health Care System or local community physicians are recruited through several sources: 1) advertisements on the website of the UNC Center for Functional GI and Motility Disorders ; 2) newspaper advertisements; 3) an existing IBS patient registry; 4) posters and brochures; and 5) mass e-mailings to UNC list-serves. Additionally, physicians are asked to inform patients with IBS about the study, describing it as a psychological support intervention to be carried out in addition to their usual conventional therapy for IBS. Study participants are remunerated for completing the assessments and as well as for completing the assigned intervention (defined as attendance at a minimum of six of the nine sessions).

### Screening, consent, and enrollment

Prospective subjects are screened by telephone by the study coordinator. If an individual is eligible for the study and wishes to participate, the study coordinator schedules a meeting at a mutually convenient time and location to explain the study, obtain informed consent, and complete initial assessments. A psychosocial assessment and gastrointestinal medical questionnaire is completed at this visit or shortly thereafter. Subjects are told that they may have to wait a short time for classes to begin while additional subjects are being recruited. Classes are scheduled to begin when a minimum of 6 subjects have been randomized to each of the two groups.

### Randomization

Upon completion of the baseline interview and questionnaires, eligible women are randomized by computer to the Mindfulness Program or IBS Support Group. This process eliminates experimenter bias in group assignment. The computer program uses a random number generator and is programmed to insure equal numbers of subjects in each arm of the study within a variable block size of 4–8 subjects. The computer program generates a treatment assignment and a study ID number associated with each eligible subject's name and birth date, and documents these in an uneditable form with a date stamp.

### Masking

The nature of the interventions does not allow masking of treating physicians, subjects or instructors. To minimize differences in subject expectancy, the experimental interventions are described to subjects only as two psychological interventions – a set of meditation procedures and a support group – both of which have been reported in previous studies to benefit patients with IBS. The intent is to prevent subjects from knowing which intervention the investigators believe is most likely to improve symptoms. To verify that patients assigned to the Support Group have the same expectation of benefit as patients assigned to the Mindfulness Program, all patients complete a Credibility Scale [25] after the first treatment session. This scale is presented to the subjects as an "early evaluation" scale.

Follow-up questionnaires are printed and mailed to subjects for completion. To maintain masking of staff involved in data input and analysis and to prevent bias, returned questionnaires bear only a research number.

### The Intervention Group – Mindfulness for Irritable Bowel Syndrome

The mindfulness-based stress and pain management program (Mindfulness Program) was based on the Mindfulness-Based Stress Reduction (MBSR) program developed by Jon Kabat-Zinn and Saki Santorelli at the University of Massachusetts. MBSR is a flexible program, adaptable to a variety of patients with wide-ranging symptoms [26]. The modification introduced was to emphasize symptoms and perceptions concerning IBS when teaching about mindfulness, as well as in group discussion. For example, it is suggested to participants that they might notice any sensations in the abdominal area and distinguish those sensations from thoughts *about *sensations. In so doing, the mindfulness intervention targets maladaptive cognitive coping styles such as catastrophizing, which have been shown in the literature to exacerbate irritable bowel symptoms [27,28].

In keeping with the MBSR program, the Mindfulness Program consists of one 2-hour session each week for 8 consecutive weeks plus a Saturday retreat near the end of the intervention. For the Saturday retreat setting of the Day of Mindfulness, we shortened the length of the day from six to four hours to match the length of time of the Support Group's Saturday session.

#### Class 1

The course begins with personal introductions to create group cohesion, an overview of the coming eight weeks, and a discussion of attitudes that support mindfulness. Participants are led through an experiential exercise that focuses on the immediacy of physical sensations and differentiates sensations from thoughts *about *sensations; they are encouraged to take this practice home as mindful eating. They are introduced to diaphragmatic breathing. Participants are then led through a lengthy scan of body sensations to facilitate non-judgmental awareness; this exercise is also assigned as part of routine home practice, along with weekly readings of relevant chapters in *"Full Catastrophe Living" *and "*IBS for Dummies."*

#### Class 2

Participants are guided through the body scan at the beginning of class. They are then encouraged to share and discuss their experiences during the preceding week, particularly with regard to home practice. Participants are introduced to sitting meditation with awareness of breathing as the primary object of attention, partially for the purpose of developing concentration. This form of meditation is added to the daily home practice, along with continuation of the body scan and new readings. The role of subjective interpretation is also presented as a way of beginning to understand personal responsibility for one's thoughts and actions. Home practice this week also includes awareness of pleasant events and of a routine daily activity.

#### Class 3

Gentle mindful yoga movements are introduced as a way of alleviating physical symptoms of stress and bringing awareness to subtle movements of the body. The discussions include the power of being in the present moment and the objective observation of thoughts as *merely thoughts*, rather than as facts or events. Home practice now includes body scan, gentle yoga, sitting meditation with focus on breathing, awareness of unpleasant events, and awareness of a different routine activity.

#### Class 4

The practice of sitting meditation is expanded to emphasize the perception of body sensations *as simply sensations *(as opposed to interpretations and thoughts *about *sensations, e.g. catastrophizing). Walking meditation is also introduced. The psychophysiology of the stress response is presented. Recommended home practice at this point consists of alternating body scan, gentle yoga, walking meditation, sitting meditation (for a longer period of time), and bringing awareness to both stress reactions and to moments of going numb or shutting down.

#### Class 5

Acknowledging the halfway point in the course, participants discuss commitment to daily practice and their experiences of the effects of the program so far. Sitting meditation is expanded to include awareness of thoughts arising and passing away, followed by a discussion of the role of mindfulness in responding to stress in everyday life. The concept of emotional intelligence is introduced. For home practice, participants are encouraged to begin experimenting with combinations of practices previously introduced that fit their needs, including body scan, gentle yoga, walking meditation, and sitting meditation. Home practice also includes a difficult communications exercise and awareness of the distinction between everyday reacting (without choice) and responding (with choice).

#### Class 6

Sitting meditation continues to lengthen and deepen via guided practice sessions that include use of metaphors that explore qualities of mindfulness. The topic of working with emotions continues from the previous class, expanding the inner resources for emotional resilience. In addition to the home practices suggested previously, participants are asked to be aware of and label their emotions for the next week.

#### Half-day Retreat

Most of the time is spent in silence, with alternating periods of sitting meditation, walking meditation, gentle mindful yoga, a body scan, and an introduction to a guided loving-kindness meditation. A discussion period at the end allows participants to share their experiences, challenges, and insights.

#### Class 7

The practice of *choiceless awareness *is introduced to the sitting meditation. Choiceless awareness is a state in which one is fully aware of the moment, yet mindfulness is not focused on any physical or mental image or object. Topics of discussion include gaining confidence in the practice and results of mindfulness, awareness as related to food and eating, and experiences in everyday life following the half-day retreat. In addition to the home practices suggested previously, attention is drawn to awareness of how we nourish our bodies, and how that relates to emotional intelligence.

#### Class 8

Class begins with a body scan, followed by sitting meditation. The discussion focuses on a brief review of the course, acknowledging where the participants are at this point in their personal practices, and cultivating resources for continued practice.

Homework assigned each week throughout the course includes daily mindfulness practices and readings from two provided texts, "*Full Catastrophe Living*" [26] and "*IBS for Dummies*" [29]. Subjects are asked to keep a daily diary, including minutes of practice time and any IBS symptoms. Subjects may choose to record diary information either electronically (submitted daily) or on paper (submitted weekly at the meetings or mailed).

### The Control Condition – IBS Support Group

We selected a social-support group intervention led by master's level social workers as the control condition. This format was selected to control for subject credibility, expectations, and attention, providing regular group contact, a group leader, and a structure similar to the Mindfulness Program group, while excluding the active elements of the Mindfulness Program. A similar control condition was used successfully in a previous study testing the effectiveness of cognitive behavior therapy for the treatment of IBS [22]. In that study, the Support Group arm was found to produce an *expectation *of benefit comparable to the cognitive behavior therapy group but less actual change in IBS symptoms than cognitive behavior therapy.

In parallel with the Mindfulness Program, the Support Group sessions consist of one 2-hour session each week for 8 consecutive weeks plus one half-day Saturday session during the second half of the intervention. The half-day session involves the preparation and sharing of an "IBS-friendly" meal by Support Group participants.

Weekly Support Group sessions focus on specific pre-designated topics and involve open group discussions about subjects' experiences with, or reaction to, the topic. Session topics, in the following order, include: (a) reasons for attending the Support Group; (b) personal experience of IBS symptoms; (c) triggers for IBS symptoms; (d) medical concerns related to IBS assessment or treatment; (e) changes in IBS symptomatology over time; (f) the impact of diet and exercise on IBS; (g) social relationships and IBS; and (h) review of the Support-Group experience.

Each session begins with an introduction of the specific IBS-related topic by the group leader. Subjects are encouraged to provide examples from their own life experiences to enhance each other's understanding of the topic. Although Support Group leaders facilitate discussion and answer questions, they do not give didactic information, behavioral prescriptions, or advice. Instead, the role of the group leader is to prompt participants with questions, facilitate discussion, and maintain an environment where disclosure is possible. To that end, leaders ask open ended questions, and promote discussion by using probes such as asking for clarification, requesting explanations, and encouraging cross-talk between group members. If subjects ask the group leader a direct question about how to manage their IBS symptoms, the group leader responds by redirecting the question to the group and urging participants to share their own coping strategies with one another.

Homework assigned each week throughout the course includes readings from the provided text, *"IBS for Dummies*" [29]. Subjects are asked to write a journal entry about the discussion topic for the coming week as it relates to their personal experience with IBS, as well as to keep a daily diary of IBS symptoms. Subjects may choose to record journal/diary information either electronically (submitted daily) or on paper (submitted weekly at the meetings or mailed).

### Outcome measures and study instruments (See Table 1)

**Table 1:** Measures for the Mindfulness and Support Group Subjects

Construct	Measure	Administration
***Primary Outcome measure***		

Symptom Severity	Irritable Bowel Symptom Severity Scale	Pre, 2 wk, 3 mo, 6 mo, 12 mo post

***Secondary Outcome measures***		

Symptom frequency	Daily Symptom Diary	Daily during the intervention and for 2 weeks post

IBS-related anxiety	Visceral Sensitivity Index	Pre, 2 wk, 3 mo, 6 mo, 12 mo post

Economic impact of IBS	The Work Productivity and Activity Impairment for IBS (WPAI: IBS)	Pre, 2 wk, 3 mo, 6 mo, 12 mo post

Co-Morbid Symptoms	Recent Physical Symptoms Questionnaire (RPSQ)	Pre, 2 wk, 3 mo, 6 mo, 12 mo post

General psychological distress	Brief Symptom Inventory (BSI)	Pre, 2 wk, 3 mo, 6 mo, 12 mo post

Anger and its expression	The State-trait Anger Expression Inventory (STAXI)	Pre, 2 wk, 3 mo, 6 mo, 12 mo post

Maladaptive coping methods	Coping Strategies Questionnaire (CSQ)	Pre, 2 wk, 3 mo, 6 mo, 12 mo post

Quality of life specific to IBS	IBS-QOL	Pre, 2 wk, 3 mo, 6 mo, 12 mo post

***Effect modification measures***		

Expectancy of success of intervention	Borkovec and Nau credibility scales	Prior to second session

Catalogue of distressing prior events	Family Inventory of Life Events (FILE)	Pre intervention only

Demographics	Age, race, education, marital status, income	Pre intervention only

***Process measures***		

Measure of mindfulness: Multiple constructs	Five Facet Mindfulness Questionnaire (FFMQ)	Pre, 2 wk, 6 mo, 12 mo post

Measure of mindfulness: Acceptance construct	Toronto Mindfulness Scale	Pre, 2 wk, 6 mo, 12 mo post

Administration of the outcome and process measures for Specific Aims 1 and 2 takes place upon entry into the study, at the completion of the 8-week programs, and again at 3, 6, and 12 months after program completion. Collection of these data requires approximately one hour of subject time at each of the 5 measurement visits.

#### Primary outcome variable

##### Irritable Bowel Symptom Severity Scale (IBSS)

The IBSS is a validated brief measure of IBS severity. Responders rate retrospectively, for the past 10 days, abdominal pain severity and frequency (separate ratings), bloating severity, dissatisfaction with bowel habits and life interference from bowel symptoms. These five ratings are totaled to obtain an overall rating of IBS severity. The IBSS has been shown to be valid and reliable [1]; it was found to discriminate IBS patients from controls, and to discriminate between patients categorized as mild, moderate, and severe by independent clinical assessment. In a study of 1603 patients with functional bowel disorders including 815 who met Rome II criteria for IBS [30], the IBSS correlated well (Spearman's rho = .66; p < .0001) with the Functional Bowel Disease Severity Index [1] and also correlated with the frequency of medical clinic visits for IBS in the prior 6 months (r = .21; p < 001). In their systematic review of outcome measures in IBS clinical trials, Bijkerk and colleagues ranked the IBSS as one of the two best outcome measures for IBS trials based on psychometric properties [31].

#### Secondary outcome variables

##### Daily Gastrointestinal Symptom Diary

The symptom diary was adapted from Blanchard et al. [32] Subjects are asked to complete daily symptom ratings on the Internet, using a password-protected web page, or on a paper version of the same diary. On separate 0–4 scales, subjects rate how much of a problem they have had in the past day with abdominal pain and tenderness, constipation, diarrhea and bloating. They are also asked to record whether symptoms interfered with eating, drinking or their usual activities, and how many minutes they did homework related to the study. A space is provided for other comments.

##### Brief Symptom Inventory-18 (BSI-18)

The BSI is an 18-item version of the Symptom Checklist-90-R (SCL-90-R) [33]. Subjects rate how much they were bothered in the last 7 days by each of 18 symptoms, including separate scores for anxiety, depression, somatization, and a global symptom severity index. The BSI has good internal consistency reliabilities (Cronbach's alpha = 0.71–0.85) and test-retest reliabilities (α = 0.68 – 0.91) as well as excellent convergent validity with the SCL-90-R (r = 0.92–0.98) [34].

##### IBS-Quality of Life (IBS-QOL)

The IBS-QOL questionnaire is a 34-item disease-specific quality-of-life scale that measures changes in physical and psychosocial functioning as a result of IBS. It is responsive to change over 12 weeks [35] and has high internal consistency (Cronbach's alpha = .95) and high reproducibility over a 7-day interval (ICC = .86) [30].

##### State-trait Anger Expression Inventory (STAXI)

This is a 44-item questionnaire that measures State Anger and Trait Anger [36]. Internal consistency for State and Trait Anger is good (α = 0.86–0.93) and it correlates well with hostility indices on the MMPI [37]. In a test of the effectiveness of an 8-week meditation intervention for patients with chronic low back pain, the State Anger measures decreased from pre- to post-intervention: F(1,15) = 3.38, p = 0.09 [38].

##### Visceral Sensitivity Index (VSI)

The VSI is a 15-item scale which measures anticipatory anxiety with respect to the likely occurrence of symptoms such as abdominal pain [39]. Internal consistency is excellent (Cronbach's α = 0.93, inter-item correlation = 0.47), and the VSI shows good convergent validity with the Hospital and Depression (HAD) Anxiety subscale (r = 0.73) and the Anxiety Sensitivity Index (ASI) (r = 0.66). Regression analysis revealed that the VSI is a strong predictor of symptom severity, accounting for 24% of the variance [39].

##### Recent Physical Symptoms Questionnaire (RPSQ)

This 26-item scale was developed by the UNC Center for Functional Gastrointestinal and Motility Disorders by systematically reviewing the literature to identify the somatic symptoms reported with increased frequency by patients with IBS [40]. Patients are asked to rate the frequency of occurrence of these symptoms in the last 30 days on a 5-point ordinal scale. The internal consistency (Cronbach's alpha) for this questionnaire is 0.86. The RPSQ correlates strongly with number of physician visits in the past year (r = .62).

##### Work Productivity and Activity Impairment for IBS (WPAI: IBS)

The WPAI: IBS measures work-time lost and activity impairment secondary to IBS. The WPAI: IBS was evaluated for accuracy and validity with 135 IBS patients in 2004 [41]. The sum for missed work time in a retrospective diary was predictive of the WPAI: IBS score. Test-retest correlations were high (r = 0.97 – 0.99) as well [41].

##### Coping Strategies Questionnaire (CSQ)

The 50-item CSQ was designed to assess coping strategies used by patients who have chronic illness [42]. The 6-item Catastrophizing subscale, the most frequently used subscale, has excellent internal consistency (alpha = .91) and correlates with measures of pain intensity and functional impairment. Three other subscales – Distraction, Ignoring Pain Sensations, and Distancing from Pain – have good internal consistency reliability (α = 0.80 – 0.83) and contain items that may distinguish between the two groups at outcome, in terms of coping styles.

#### Moderators

##### Demographics

The study is limited to women. The demographic questionnaire collects data about each woman's age, marital status, race/ethnicity, years of education, approximate family income, and medical history related to IBS.

##### Family Inventory of Life Events and Changes (FILE)

The FILE was designed to assess the accumulation of stressful events experienced by a family [43]. Reliability is well established for the full scale (α = 0.72 – 0.81), although the subscales are not as stable. Test-retest reliability for the full scale is good (r = 0.72 – 0.77).

#### Evaluation of Subject Expectation/Bias and Protocol Variability

##### Credibility scale (Attitude Towards Treatment Questionnaire)

This measure was based on the original measure by Borkovec and Nau which assesses patients' expectations of benefit once treatment has been explained [25]. The scale has been found to distinguish between standard psychotherapy approaches and illogical placebo treatments, it is predictive of clinical improvement, and it is relatively independent of symptom severity. Although originally designed to focus upon treatments for fear, Drossman, Toner, and Whitehead modified the questions to target psychological (and placebo) treatments of functional gastrointestinal disorders [44].

##### Quality Control (See Table 2)

**Table 2:** Quality Control and Feasibility Measures

Component	Measure	Responsible party
Context	Log of comments about space, contacts with other interested parties outside of participants	Instructors Study coordinator

Dose delivered	Attendance log Enrollment diary	Instructors Study coordinator

Dose received	Participant log of adherence to homework assignments	Instructors

Fidelity	Video-taping of instructors delivering interventions	Research assistant, PI

Instructors record attendance, logistical problems and other study-related issues. The study coordinator keeps a similar log of logistical issues and contacts subjects not completing the intervention to evaluate factors leading to dropout. To evaluate subject engagement, subjects are prompted to keep a log of their homework practice in their daily diaries.

To evaluate intervention fidelity, we measure instructor adherence to protocol through videotaping the second and seventh Mindfulness Program and IBS Support Group sessions with an unmanned stationary video recorder. Video-taping is for purposes of identifying variations in instructor attitude, communication bias and systematic variations in behavior between treatment groups. It is also used to assess adherence to protocol as well as to screen for any group-dependent differences in content or style of communication between the instructor and participants.

#### Process Measures

##### Five Facet Mindfulness Questionnaire (FFMQ)

The FFMQ was developed by Baer based on exploratory and confirmatory factor analyses of five existing questionnaires designed to explore constructs of mindfulness identified in prior research [45]. The resulting instrument is a 39-item, Likert-type measure assessing five identified facets of mindfulness: non-reactivity to inner experience; non-judging of inner experience; acting with awareness; describing; and observing. Individual facets of the FFMQ correlate positively with openness to experience, emotional intelligence, and self compassion, and negatively with alexethymia, dissociation, and psychological distress [45].

##### Toronto Mindfulness Scale

The Toronto Mindfulness Scale adapted by J. Kristeller measures the acceptance construct in mindfulness. The scale is currently undergoing reliability and validity testing [23].

#### Adverse events

All subjects are asked to record any adverse events in their diaries and to report them to the group leaders, the study coordinator or the study physician. In addition, they are asked about their experiences weekly at the training sessions. Subjects are also strongly encouraged to discuss with the research staff anything they feel to be an adverse event or other issues that are problematic for them about the study.

### Analytic strategy

#### Sample Size and Power Calculation

Outcome measures for clinical trials were investigated, including a report of adequate relief of IBS symptoms and the magnitude of symptom reduction on the IBSS, our primary outcome in this trial [46]. Based on this data, patients reporting "adequate relief," defined here as a clinically meaningful outcome, showed a reduction in IBSS scores of 87 units. The power to detect this change at α = .05 in samples of 30 per group is approximately 84%. In the current study, 45 subjects per group is planned.

Regression modeling will be used to test the primary hypothesis. A simple linear regression model with the IBSS score at the end of the study controlling for IBSS score at the baseline will be used to test for significant treatment effects. We will use repeated measures analysis on IBSS measures to detect whether the change in IBSS scores differ significantly between the two groups. Because of the correlated nature of the repeated measures, regression models using a generalized estimating equations (GEE) approach will be used to test the hypothesis of change. A significant time and treatment interaction will detect significant changes in the score. A similar analysis strategy using GEE methods will be used to analyze daily records of IBS symptoms. All data analysis will be performed on an intent-to-treat basis. The GEE method assumes that missingness in data are completely at random. Under the missing at random assumption, it is possible to conduct analyses using multiple imputation procedures [47].

## Discussion

Recruitment into the study has proceeded as planned, with subjects being recruited via the UNC Center for Gastrointestinal Disorders Subject Registry, as well as through flyers, email announcements, and newspaper advertisements. At present, 214 individuals have been contacted to screen for eligibility. Of those, 127 did not qualify: 66 were ineligible (previous meditation training, non-IBS bowel disease, psychiatric disorder, diabetes, and lactose intolerance), 32 declined participation (in the area only temporarily, moved away, incompatible schedule, symptom free now), and 29 asked to be contacted later.

Eighty-seven subjects have been enrolled so far, of whom 21 withdrew (14 in Mindfulness/7 in Support group): 10 withdrew before attending a class and 11 others withdrew after one or more classes. Reasons for withdrawal included pregnancy, family illness, new medication, schedule changes, and moving). Sixty-six have completed or are in the process of completing the study. There have been no reported adverse events related to the study.

This report endorses the feasibility of undertaking a rigorous randomized clinical trial of mindfulness training for people with IBS, using a standardized MBSR protocol adapted for those experiencing IBS, compared to a control social-support group previously utilized in IBS studies and adapted here.

Final analysis of the data is not yet available for comparing the two intervention groups in terms of protocol credibility, satisfaction, or the primary and secondary outcomes. However, several preliminary conclusions can be made at this point.

### Feasibility

Although recruitment has been challenging, our primary aim of proving feasibility has been achieved. Subjects completed the study measures in a timely fashion with minimal assistance and fairly good compliance. Subject diaries have been a rich source of information and future studies will put more emphasis on diary compliance. Other aims, including acquisition of secondary outcome measure data and mindfulness measurement assessment tools, have also been achieved. Recruitment will continue through June 2009, with data analyses to follow.

### Recruitment challenges

Both interventions require subjects to commit to attending nine group sessions over a period of eight weeks. The busy lives of many women, especially those with chronic conditions such as IBS, may preclude their making such a long commitment. In addition, competing IBS studies at UNC with shorter time commitments and higher compensations have made this opportunity less appealing to some. Despite these challenges, significant efforts on the part of the research staff and our research fellows have ensured that adequate numbers of subjects have been available for study.

### Group Assignment and Credibility

A large majority of participants in both groups have expressed satisfaction with their group assignment and the drop-out rate has been reasonably low after exposure to the two interventions. Preliminary assessment of intervention credibility reveals that there is no major barrier to completion of the study for members of either group.

### Minority Recruitment

We have had success in recruiting minorities for the study (approximately 19% African-American, 2% Hispanic/Latina). Since the study requires participants to communicate adequately in English in group discussions, we have not targeted those who do not speak English.

### Study Benefits

The benefits to subjects may be significant. First, the symptoms experienced by the subjects may improve significantly as a direct result of being part of the study. All subjects undergo an evaluation of their IBS symptoms, coping strategies and psychological factors that influence the course of their condition. Prior research suggests that mindfulness training may be useful as adjunctive treatment for a number of disorders associated with IBS that may be present in members of the study cohort. Subjects in both groups may experience greater clinical improvement than they might otherwise experience with usual medical care alone. The process of participating in the Mindfulness Program or the Support Group could enhance self-efficacy. Participants may learn improved methods of managing IBS from each other's experience.

Results of this project will provide important information, in addition to addressing the feasibility of conducting a larger study using this methodology, specifically relating to issues of integration of mindfulness meditation with conventional medical care; application of mindfulness for IBS; use of a support-group control; and statistical approaches as described in the Methods/Design section. Knowledge gained about issues of control therapies in CAM research, subject recruitment and measurement methods may be of broader significance to the development of clinical trials using other CAM modalities.

We will also gain evidence for or against the integration of mindfulness with usual medical care for treatment of a common and debilitating disease. Results supporting the use of mindfulness will lead to expanded studies of this modality, powered to define more clearly the magnitude of benefits and the scope of its clinical uses. If the use of mindfulness for IBS is supported by future studies, mindfulness-based treatment of IBS could potentially diminish IBS patients' conventional health care utilization, with its associated side effects and substantial costs.

Additionally, a well-conceived and adequately powered clinical trial based on our study showing positive results could further elucidate the mechanism of action of mindfulness, which remains largely putative at this point. Knowledge derived from this and follow-up studies could enhance our understanding of human psychobiology from the standpoint of health maintenance and healing in ways that have not been fully appreciated or conceptualized to date.

By clarifying methodological issues, such as subject accrual, design of control interventions, effect size estimation, and whether a definitive trial is even warranted, the results of this study may help others conducting research in mind-body medicine to improve the nature and quality of their research and open the way for future definitive studies.

## Competing interests

The authors declare that they have no competing interests.

## Authors' contributions

Authors SG and WEW were involved in developing the original idea for funding. SG was PI, and WEW, co-PI, on the funded proposal, with JDM and OP as co-investigators on the proposal. OP is providing oversight of data collection and statistical assessments, while JDM is involved in subject screening, adverse events, data interpretation and manuscript preparation. RC is study coordinator. KRF has assisted with the data protocol development and management. WF and ELG have been particularly involved in refining the intervention protocols. All authors participated in the design of the study and development of research protocols. All authors contributed to and approved the final manuscript.

## Pre-publication history

The pre-publication history for this paper can be accessed here:


